# Reduced Fetal Telomere Length in Gestational Diabetes

**DOI:** 10.1371/journal.pone.0086161

**Published:** 2014-01-22

**Authors:** Jian Xu, Junyi Ye, Yanting Wu, Hong Zhang, Qiong Luo, Cong Han, Xiaoqun Ye, Hanzhi Wang, Jing He, Hefeng Huang, Yun Liu, Minyue Dong

**Affiliations:** 1 Women's Hospital, School of Medicine, Zhejiang University, Hangzhou, Zhejiang Province, China; 2 Key Laboratory of Reproductive Genetics, Ministry of Education, Zhejiang University, Hangzhou, Zhejiang Province, China; 3 Institutes of Biomedical Sciences, Fudan University, Shanghai, China; 4 Key Laboratory of Molecular Medicine, The Ministry of Education, Department of Biochemistry and Molecular Biology, Fudan University, Shanghai, China; Université de Montréal, Canada

## Abstract

Gestational diabetes mellitus (GDM) is an important complication of pregnancy that poses significant threats to women and their offspring. Telomere length shortens as cellular damage increases and is associated with metabolic diseases. Telomere length in fetal leucocytes was determined in 82 infants of women with GDM (N = 82) and 65 normal pregnant women (N = 65). Women with preeclampsia (N = 45) and gestational hypertension (N = 23) were also studied. In the GDM group, telomere length was significantly shorter than normal pregnancy (P = 0.028), but there were no significant differences in fetal telomere length between preeclampsia and normal pregnancy (P = 0.841) and between gestational hypertension and normal pregnancy (P = 0.561). Regression analysis revealed that fetal telomere length was significantly associated with intrauterine exposure to GDM (P = 0.027 after adjustment for maternal age, gestational age at delivery, birth weight and fetal gender). Shortened telomere length may increase the risk of metabolic diseases in adulthood of GDM offspring.

## Introduction

Telomeres contain noncoding hexameric tandem repeats ranging from a few to 15 kilobases in length that maintain chromosomal stability and genomic integrity [1], [2]. Telomeres shorten with cell division and telomere function depends on both a minimal length of TTAGGG repeats and telomere-binding proteins [1], [2]. Telomeres shorten with age in most somatic tissues [3]. Telomere shortening is also influenced by genetic factors and epigenetic regulation, as well as pro-inflammatory and oxidative stress, while the ability of telomerase to counteract these influences is limited [1], [2], [4].

Telomere length, a heritable human trait, has been considered a marker of cumulative cell damage [1], [2]. And, shortening of the length of telomeres is associated with a series of metabolic abnormalities including impaired glucose tolerance, dyslipidemia, obesity, diabetes and cardiovascular diseases. Leukocyte telomere length (LTL) reflects the replication of hematopoietic stem cells that are sensitive to adverse external conditions; it is a biomarker for the evaluation of ageing and past exposure to adverse conditions [3], [5], [6], [7].

Gestational diabetes mellitus (GDM) poses both short-term and long-term risks to the health of women and their offspring [8], [9]. The offspring of women with GDM are at increased risk of metabolic diseases including obesity, diabetes and cardiovascular diseases [8], [9]. Modifications in epigenetic and endocrine regulation are among the suggested mechanisms linking intrauterine exposure to GDM with metabolic diseases in adulthood [10]. However, the mechanisms of this increased risk of chronic diseases induced by intrauterine exposure to GDM remain largely unknown. Similarly fetuses exposed to maternal preeclampsia carry an increased risk of cardiovascular disease in later life through unexplained mechanisms [8], [10], [11]. Telomere biology may offer a new route for exploring the adverse health effects of intrauterine exposure to GDM and preeclampsia [12], [13], [14], [15].

In the current investigation, we hypothesized that maternal GDM or preeclampsia, posed adverse conditions to the fetus that may induce fetal telomere attrition. To verify this hypothesis, we determined cord blood leukocyte telomere length in women with GDM, preeclampsia, gestational hypertension and normal pregnancy with a quantitative poly-chain reaction (PCR)-based assay.

## Materials and Methods

### Subjects

Women with GDM (N = 82), gestational hypertension (N = 15), preeclampsia (N = 45) and normal pregnancies (N = 65) were consecutively recruited to this cross-sectional survey. Pregnancy was diagnosed by positive human chorionic gonadotropin (hCG) test. Gestational age was calculated by ultrasound examination in the first trimester. GDM was diagnosed according to the criteria recommended by International Association of Diabetes and Pregnancy Study Groups (IADPSG) [16], and gestational hypertension and preeclampsia were diagnosed and classified according to the criteria recommended by American college of Obstetrics and Gynecology (ACOG) [17]. Women in the control group had no gestational complications and gave birth to healthy neonates of appropriate size for gestational age. To exclude the possible effect of labor on fetal LTL and for the feasibility of sample collection, only women delivered by elective Cesarean section were included.

Maternal age was significantly different between the groups (F = 18.96, P<0.001) (Table 1). Women with GDM, gestational hypertension or preeclampsia were significantly older (P<0.001) than those with normal pregnancy. There was a significant difference in gestational age at delivery (F = 55.41, P<0.001). Gestational age was significantly lower (P<0.001) in preeclampsia compared to normal pregnancy, pregnancy complicated by GDM or gestational hypertension. Neonatal birth weight was also significantly different (F = 23.86, P<0.001). Neonatal birth weight was significantly higher (P<0.001) in GDM compared to normal pregnancy, gestational hypertension or preeclampsia, but was significantly lower (P<0.001) in preeclampsia compared to normal pregnancy, GDM or gestational hypertension while it was not significantly different between normal pregnancy and gestational hypertension (P = 0.381). There was no significant difference in percentage of male or female fetuses among normal pregnancy, GDM, gestational hypertension and preeclampsia (*x* ^2^ = 1.563, P = 0.668).

**Table 1:** Clinical data.

	Normal pregnancy	Gestational diabetes	Gestational hypertension	Preeclampsia	
N	65	82	15	45	
Maternal age (y)	28.6±3.4	30.61±3.8*	31.8±4.7*	30.8±5.0*	F = 18.96 P<0.001
Gestational age at delivery (w)	38.68±1.36	38.29±1.90	38.64±1.15	33.40±3.71**	F = 55.41 P<0.001
Birth weight (g)	3263±395	3629±543***	3202±686	2533±739**	F = 23.86 P<0.001
Fetal gender	Male: 32; Female: 33	Male: 44; Female: 38	Male: 10; Female: 5	Male: 23; Female: 22	*x* ^2^ = 1.563 P = 0.668

* P<0.001 vs normal pregnancy.

** P<0.001 vs normal pregnancy, gestational diabetes and gestational hypertension.

*** P<0.001 vs normal pregnancy, gestational hypertension and preeclampsia.

Exclusion criteria included multiple gestation, pre-gestational diabetes mellitus, chronic hypertension, infectious diseases recognized in pregnancy, premature rupture of membrane, active labor, polyhydramnios and concurrent medical complications. This cross-sectional study was approved by the Ethics Committee of Women's Hospital, School of Medicine, Zhejiang University and informed written consents obtained from all participants.

### Sample collection

Cord blood samples were collected in EDTA-treated tubes at delivery. Lymphocytes were isolated from cord blood and stored at −80C before extraction of DNA. Total DNA was isolated from lymphocytes using buffer ATL, proteinase K, and RNase A (Qiagen, Inc., Valencia, CA) followed by phenol–chloroform extraction and ethanol precipitation.

### Measurement of leukocyte telomere length (LTL)

Telomere length was measured using an established and validated quantitative PCR-based technique [18]. The relative telomere length was calculated as the T/S (telomere/single copy) ratio using RNase P as a reference. The quantities of telomere repeats and RNase P were also determined for each sample in duplicate in a 10 μl reaction mixture in the same microplate in an ABI Applied Biosystems 7900 HT Thermal Cycler (Applied Biosystems, Foster City, California).

The telomere reaction mixture contained 1x SYBR green TaqMan Gene Expression master mix (Applied Biosystems, Foster City, California), 1 ng of DNA, 300 nM Tel-F and Tel-R primers (Tel-F: 5′-CGGTTTGTTTGGGTTTGGGTTTGGGTTTGGGTTTGGGTT-3′; Tel-R: 5′-GGCTTGCCTTACCCTTACCCTTACCCTTACCCTTACCCT-3′). A commercial kit was used according to the manufacturer's instructions to estimate the level of RNase P gene expression as an internal standard (TaqMan RNase P Detection Reagents Kit, Applied Biosystems, Foster City, California) using 1x primers and the TaqMan probe reagent, 1x TaqMan Genotyping Master mix and 3 ng of DNA. The cycling conditions for the telomere and RNase P assays were as follows: 95°C for 10 min, followed by 50 cycles of 95°C for 15 sec and 60°C for 1 min.

Along with the samples, each assay contained a calibrator sample (DNA from pooled samples). Dilution series (0.675-5 ng in two-fold dilutions) were assayed for both telomere and RNase P to establish the linear range. Good linearity was observed across this range (R^2^>0.97). Any samples outside this range were diluted and rerun. For quality control, all samples were run in duplicate and duplicate values were checked for correlation. Samples showing a coefficient of variation (CV) >2% were excluded and rerun. To test the reproducibility of the assay, multivariate samples were randomly chosen and run again, and a high level of agreement was observed between the T/S ratios from the 2 runs (R^2^ = 0.764, P<0.0001).

### Statistical analysis

Data were tested by Kolmogorov–Smirnov test for their distribution. Normally distributed data were presented in mean ± standard deviation (SD) and compared by Student's t test or one-way ANOVA with LSD multiple comparisons. Regression was used to analyze the relationship of fetal telomere length with maternal age, gestational age at delivery, neonatal birth weight and fetal gender. Statistic package SPSS (Statistical Analysis System, Chicago, IL, USA) was used for data analysis. A P<0.05 was considered to be statistically significant.

## Results

**Figure 1:**
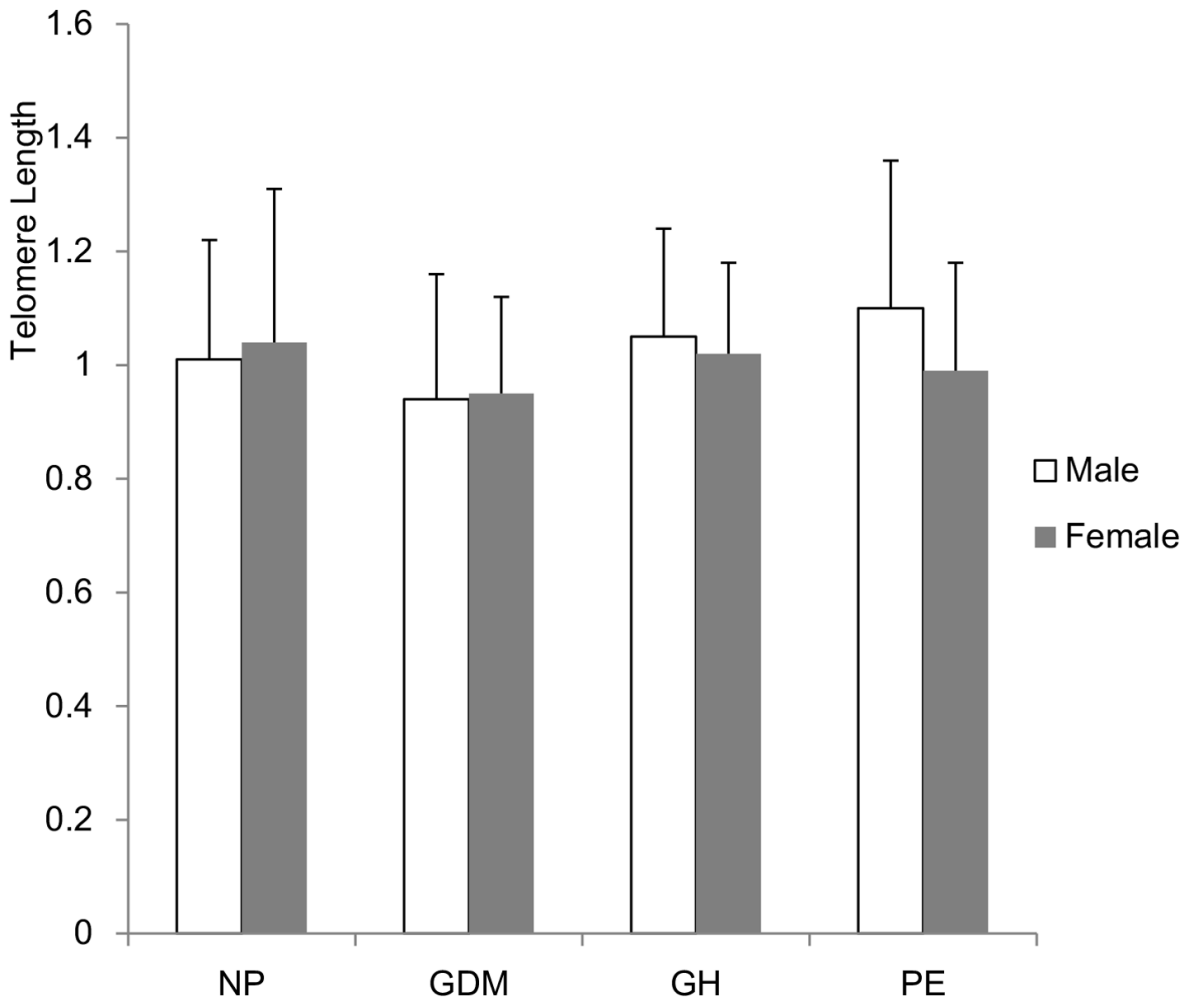
Comparison of telomere length between male and female infants. Telomere length was not significantly different between male and female fetuses of women with normal pregnancy (NP) (P = 0.587), gestational diabetes (GDM) (P = 0.746), gestational hypertension (GH) (P = 0.772) or preeclampsia (PE) (P = 0.107).

**Figure 2:**
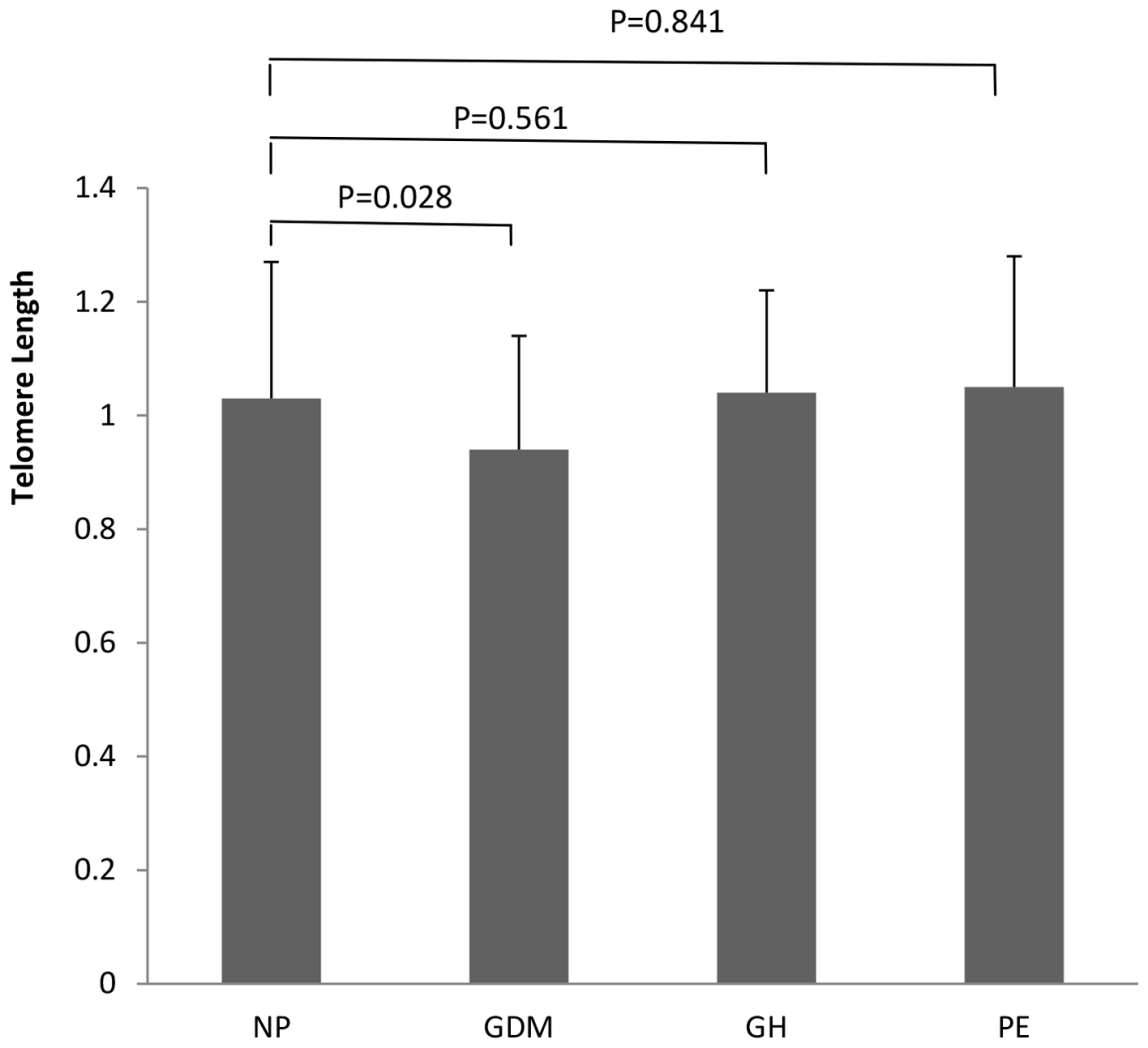
Comparison of fetal telomere length among normal pregnancy (NP), gestational diabetes (GDM), gestational hypertension (GH) and preeclampsia (PE). There were significant differences in fetal telomere length (F = 3.083, P = 0.028). Fetal telomere length was significantly shorter in gestational diabetes than that of normal pregnancy (P = 0.028). There was no significant differences in fetal telomere length between normal pregnancy and gestational hypertension (P = 0.561), between normal pregnancy and preeclampsia (P = 0.841).

**Figure 3:**
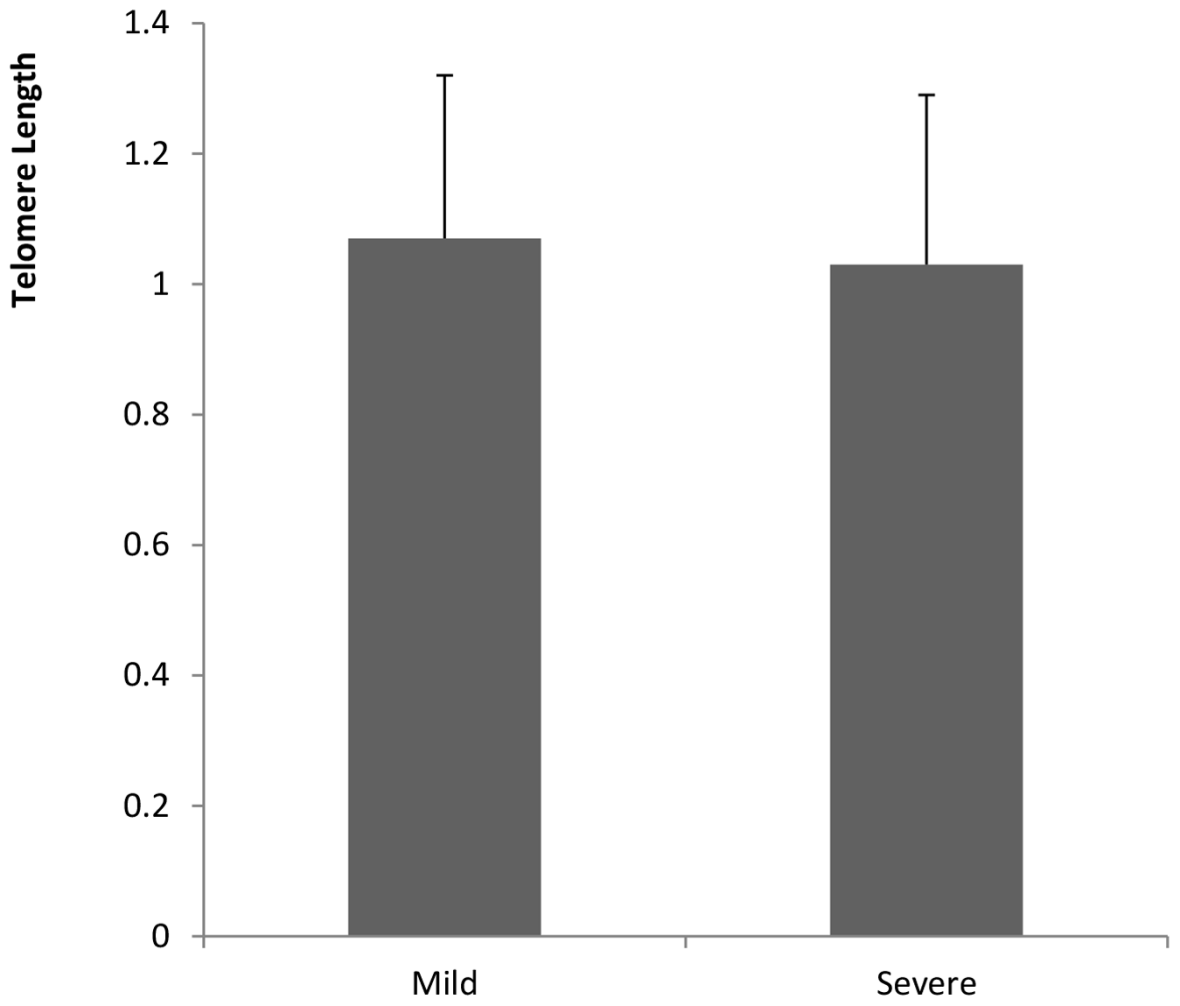
Comparison of fetal telomere length between mild and severe preeclampsia. Telomere length was not significantly different in fetuses of women with mild and severe preeclampsia (P = 0.726).

There were no significant differences in telomere length between male and female fetuses of women with normal pregnancy (*t* = 0.546, P = 0.587), GDM (*t* = 0.325, P = 0.746), gestational hypertension (*t* = 0.295, P = 0.772) or preeclampsia (*t* = 1.647, P = 0.107) (Figure 1). Thus data of male and female fetuses were pooled. There were significant differences in telomere length among fetuses of women with GDM, gestational hypertension, preeclampsia and normal pregnancy (F = 3.083, P = 0.028) (Figure 2). The telomere length was significantly shorter in fetus of GDM women than that of normal pregnancy (P = 0.028), but there were no significant differences in fetal telomere length between gestational hypertension and normal pregnancy (P = 0.561) and between preeclampsia and normal pregnancy (P = 0.841). Telomere length was not significantly different in fetuses of women with mild and severe preeclampsia (t = 0.459, P = 0.726) (Figure 3).

Multivariate regression analysis revealed that fetal telomere length was significantly associated with intrauterine exposure to GDM (R^2^ = 0.019, P = 0.027 after adjustment for maternal age, gestational age at delivery, birth weight and fetal gender) but not maternal age, gestational age at delivery, birth weight or fetal gender. To evaluate the effect of maternal age on fetal telomere length, linear regression was performed between maternal age and fetal telomere length. Fetal telomere length was not significantly correlated with maternal age in normal pregnancy (R = 0.081, P = 0.532), GDM (R = 0.058, P = 0.606), gestational hypertension (R = 0.132, P = 0.652), preeclampsia (R = 0.017, P = 0.923), or all subjects (R = 0.029, P = 0.684).

## Discussion

In the current investigation, we found that telomere length was significantly shortened in fetuses of GDM women as compared with fetuses of normal pregnant women. Our findings indicate that GDM may have an impact on telomere biology. Since shortened telomere length is associated with increased risks of cardiovascular diseases, hypertension, obesity and diabetes (metabolic diseases), fetal telomere shortening may be among the mechanisms linking maternal GDM with increased risks of metabolic diseases in offspring. However, our findings were different from those of Cross et al [13]. They found that the telomere length was not significantly different in the offspring of women with type-1 diabetes, type-2 diabetes, or gestational diabetes from their controls but telomerase activity was significantly enhanced in the GDM offspring when compared with controls.

The intrauterine milieu of GDM exposes the fetus to oxidative stress and is possibly involved in fetal telomere attrition. Kamath et al [19] found markedly increased lipid peroxidation and protein oxidative damage in the erythrocytes of infants born to mothers with GDM. Similarly, Kinalski et al [20] showed that the level of malondialdehyde (MDA, a product of lipid peroxidation) was increased while superoxide dismutase (SOD, a scavenging enzyme against lipid peroxidation) activity decreased in cord blood of infants from mothers with GDM. Furthermore, Sobki et al [21] observed a significant depletion of a-tocopherol, an anti-oxidant, in the cord blood of GDM fetus, which is indicative of a possible oxidative stress in fetuses of GDM women.

On the other hand, our findings that telomere length did not differ in fetuses of women with preeclampsia or gestational hypertension from controls confirmed previous observations. Okuda et al [15] found that telomere length as measured by the mean length of the terminal restriction fragments (TRF) in samples of white blood cells, umbilical artery and skin was not significantly different between newborns of mothers complicated with hypertension during pregnancy, preeclampsia and newborns of mothers without those complications. Akkad et al [12] determined the telomere length in small-for-gestational-age (SGA) infants who are exposed to an intrauterine milieu of under-nutrition and hypoxia. Similarly infants exposed to maternal preeclampsia showed no significant difference in telomere length between SGA infants and controls.

A small number of studies looked at the effects of GDM or preeclampsia on maternal and placental telomere length. Harville et al [22] reported that there was no association of maternal telomere length with pre-eclampsia, and shorter maternal telomeres was possibly associated with GDM, but differences might be due to chance. Biron-Shental et al [23] found that telomere length was significantly shorter in preeclampsia, intrauterine growth restriction, and preeclampsia plus intrauterine growth restriction placentae while telomerase reverse-transcriptase was significantly higher in controls compared with the other groups. These observations suggest that telomere biology may participate in the pathogenesis of these complications.

Sample sizes were similar in our study and that of Cross et al [13] while the populations where the studies were conducted and the methods used to determine telomere length were different. A quantitative PCR-based method was used in our study while an FCM-based method was used in the experiment of Cross et al [13]. Subjects were all Chinese Han people in our cohort while predominantly white in that of Cross et al [13]. We cannot determine the reasons for the different results, however, we cannot completely exclude the possible effects of these differences in subjects and methods.

Telomere shortening is caused by disturbances during cell division, and oxidative stress is the primary reason for telomere shortening. Conflicting results on oxidative stress status in fetuses born to preeclamptic women were described. Fetal oxidative stress status was reported to be increased [24], unchanged [25], and even decreased [26]. In contrast, the reports on fetal oxidative stress status in GDM were quite consistent and oxidative status was also enhanced in fetuses of diabetic women [27]. The difference in the oxidative stress status fetuses of preeclamptic women and GDM women may be the reason, at least among the reasons, for different telomere lengths. Absence of data regarding oxidative stress in the current investigation may have contributed to the different results.

In summary, shorter telomere length was demonstrated in infants born to GDM women, which indicates intrauterine exposure to GDM enhances fetal telomere attrition and shortened fetal telomere length may be among the mechanisms linking maternal GDM with increased risk of metabolic diseases in offspring. However, the role of shortened telomere length in the development of adult diseases needs further investigation.
